# Mapping of infection prevention and control education and training in some countries of the World Health Organization’s Eastern Mediterranean Region: current situation and future needs

**DOI:** 10.1186/s13756-023-01299-9

**Published:** 2023-09-04

**Authors:** Rima Moghnieh, Amal Saif Al-Maani, Jana Berro, Nour Ibrahim, Rana Attieh, Dania Abdallah, Jameela Al-Ajmi, Dhouha Hamdani, Najiba Abdulrazzaq, Abeer Omar, Safa Al-Khawaja, Rami Al-Abadla, Salam Al-Ratrout, Mohammad Gharaibeh, Zakaria Abdelrahim, Hiba Azrag, Karima Mayar Amiri, Atika Berry, Bashar Hagali, Jamal Kadhim, Huda Al-Shami, Mumtaz Ali Khan, Roula Husni, Iman Heweidy, Bassim Zayed

**Affiliations:** 1 Department of Internal Medicine, Lebanese American University Medical Center-Rizk Hospital, Beirut, Lebanon; 2 Senior Consultant in Pediatric Infectious Diseases & Infection Prevention & Control, Directorate General for Disease Surveillance & Control, Ministry of Health, Muscat, Oman; 3 Gilbert and Rose-Marie Chagoury School of Medicine, Lebanese American University, Beirut, Lebanon; 4 Pharmacy Department, Makassed General Hospital, Beirut, Lebanon; 5 Senior Consultant Infectious Diseases, Executive Director Infection Prevention & Control, Ministry of Public Health, Doha, Qatar; 6 Infection Control Specialist, Quality Improvement & Patient Safety, Ministry of Public Health, Doha, Qatar; 7 Al Baraha Hospital, Ministry of Health and Prevention, Dubai, United Arab Emirates; 8 Surveillance Department, Infection Control Directorate, National Focal person of AMR and Head, Ministry of Health, Kuwait City, Kuwait; 9 Department of Internal Medicine, Salmaniya Medical Complex, Ministry of Health, Manama, Kingdom of Bahrain; 10 Infection Control Consultant, Director of Safety & Infection Prevention & Control Unit, Gaza, Palestine; 11 Quality & Patient Safety Unit Director, The Westbank, Palestine; 12 Infection Prevention & Control Program, Ministry of Health, Amman, Jordan; 13 Infection Preventionist, Infection Control Office, Jordan University Hospital, Amman, Jordan; 14 Development and Accreditation, Federal Ministry of Health, Khartoum, Sudan; 15 General Directorate of Curative Medicine, Ministry of Public Health, Kabul, Afghanistan; 16 Department of Preventive Medicine, Ministry of Public Health, Beirut, Lebanon; 17 General Assembly of Damascus Hospital, Ministry of Health, Damascus, Syrian Arab Republic; 18 Infection Prevention & Control Program, Ministry of Health, Baghdad, Iraq; 19 Infection Prevention & Control Program, Ministry of Health, Sanaa, Yemen; 20 Centers for Disease Control, NIH and Coordinator FELTP, Islamabad, Pakistan; 21 Antimicrobial Resistance/Infection Prevention & Control Unit, Infection Prevention & Control Consultant, Eastern Mediterranean Regional Office, World Health Organization, Cairo, Egypt; 22 Department of Communicable Disease, Antimicrobial Resistance/Infection Prevention & Control Unit, Eastern Mediterranean Regional Office, World Health Organization, Cairo, Egypt; 23 Department of Internal Medicine, Division of Infectious Diseases, Lebanese American University Medical Center, Beirut, Lebanon

**Keywords:** Infection prevention and control, Education, Training, World Health Organization, Eastern Mediterranean Region

## Abstract

**Background:**

A strong understanding of infection prevention and control (IPC) procedures and comprehensive training among healthcare workers is essential for effective IPC programs. These elements play a crucial role in breaking the chain of nosocomial infections by preventing the transmission of resistant organisms to patients and staff members. This study mapped the components of IPC education and training across various member states of the World Health Organization (WHO) in the Eastern Mediterranean Region (EMR) at national, academic, and healthcare institutional levels.

**Methods:**

A self-administered structured online questionnaire based on the WHO “Core Component 3” of IPC programs at the national and acute healthcare facility levels (IPC education and training) was given to national IPC focal persons in each of the WHO’s EMR countries between February and March 2023.

**Results:**

From 14 of the 22 countries,15 IPC persons participated in the survey. Most countries have scattered nonhomogeneous IPC education programs in human health undergraduate majors without considering it a standalone module. Academic institutions are rarely involved, and elaborate and predefined undergraduate IPC education programs provided by universities are present in 21.4% of the countries. In 71.4% of these countries, postgraduate training targeting IPC professionals is provided by national IPC teams, primarily based on national IPC guidelines developed with the aid of the WHO. Generally, healthcare worker training relies heavily on healthcare facilities in 92.9% of the countries, rather than on a national training program. In 42.9% of the countries, practicing IPC physicians are not necessarily specialists of infectious disease or medical microbiologists and IPC nurses are not required to specialize in IPC. However, nonspecialized IPC professionals are expected to undergo training upon employment and before beginning practice. Nongovernmental organizations such as the WHO play a significant role in IPC education and in supporting national IPC authorities in establishing national IPC guidelines, as it is the case in 78.6% of these countries.

**Conclusion:**

Clear disparities exist in IPC education and training across different countries in the WHO’s EMR. Establishing a regional scientific network specializing in IPC would help bridge the existing gaps and standardize this education within individual countries and across countries in the region. This region needs to establish IPC certification standards and standardized education curricula.

**Supplementary Information:**

The online version contains supplementary material available at 10.1186/s13756-023-01299-9.

## Introduction

During the first quarter of the 21st century, the world has witnessed major outbreaks and epidemics, such as those caused by the 2009 influenza A disease, the Ebola virus disease, the Middle East respiratory coronavirus syndrome, and most recently, the coronavirus disease 2019 (COVID-19) and the 2022 monkeypox virus. These health emergencies have highlighted gaps in infection prevention and control (IPC) programs at both national and facility levels worldwide, regardless of countries’ economic development or available resources [1]. Additionally, the pandemic of multidrug- and extensively drug-resistant organisms causing difficult-to-treat healthcare-associated infections (HAIs) is equally challenging global healthcare systems [2]. Antimicrobial resistance (AMR) has been slowly and silently growing, and the true scale of this health crisis has been difficult to understand or quantify [2].

However, there is no doubt that more than half of HAIs can be prevented by scaling up a range of effective IPC interventions [3]. The World Health Organization (WHO) has developed a set of recommendations known as the core components of effective IPC programs [3]. These components are derived from evidence-based conclusions regarding their effectiveness in curbing HAI, expert opinions, and the experience of key stakeholders in the field [3]. The core components at the national and facility level are (1) IPC programs; (2) IPC guidelines; (3) IPC education and training; (4) HAI surveillance; (5) multimodal improvement strategy implementation of IPC programs; and (6) IPC monitoring, auditing, and feedback at the national and facility levels. Additional components at only the facility level include (7) workload, staffing, and bed occupancy and (8) the built environment, materials, and equipment for IPC [3].

**Fig. 1:**
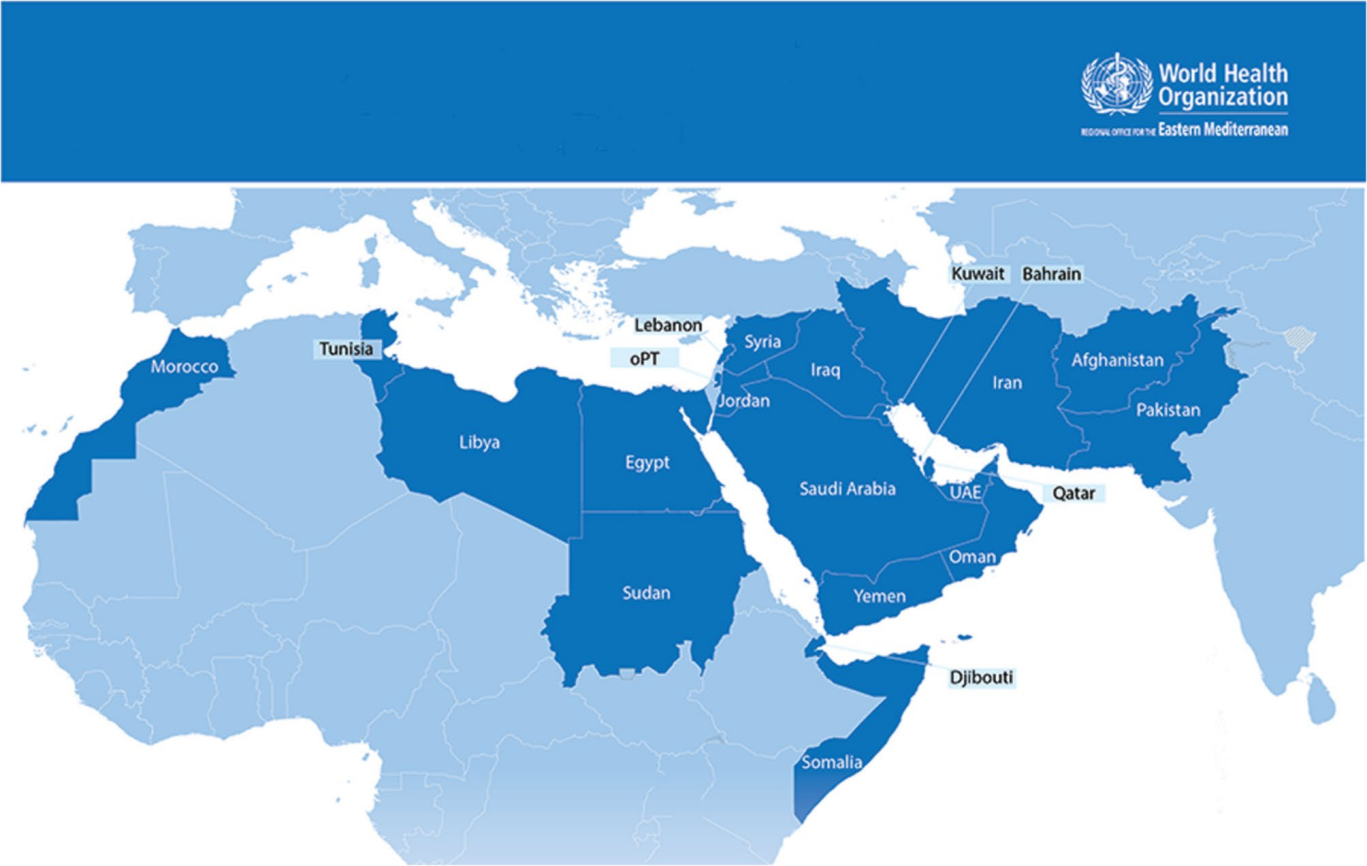
Geographical Map of the World Health Organization (WHO)-Eastern Mediterranean Region (EMR) Countries N.B. WHO-EMR countries include Afghanistan, Bahrain, Djibouti, Egypt, Iran (Islamic Republic of), Iraq, Jordan, Kuwait, Lebanon, Libya, Morocco, Occupied Palestine Territory (OPT), Oman, Pakistan, Qatar, Somalia, Sudan, Syrian Arab Republic, Tunisia, United Arab Emirates (UAE), and Yemen)

In 2022, the WHO published its first global report on IPC, providing a situational analysis of how IPC programs are implemented at the national and facility levels in countries worldwide [1]. The report addressed the impact and cost-effectiveness of IPC practices and emphasized strategies to support countries in improving their IPC programs as part of a high-priority health agenda [1]. The WHO’s global report on IPC also separately discussed the situation in the WHO’s Eastern Mediterranean Region (EMR), which comprises 21 member states and the occupied Palestinian territories with a population of nearly 745 million (Fig. 1). Although these countries share Arabic as a common official language, they are culturally, ethnically, and economically heterogeneous. According to 2021 World Bank data, 6 high-income, 11 middle-income, and 5 low-income countries form the WHO’s EMR. The aforementioned recent epidemics and outbreaks have exposed gaps in this region’s IPC programs. The WHO’s 2021–2022 global survey on IPC minimum requirements at the national level in the EMR revealed that 68% of countries had an active national IPC program and 53% had an appointed IPC-trained focal person [1]. Regarding the third core component of education and training, a curriculum for IPC in-service training was available in 72% of the countries and recommendations for in-service training were provided with the content and support of national IPC teams in approximately 60% of the countries [1]. However, less than 30% monitored the effectiveness of this training at least annually [1].

A strong understanding of IPC procedures and providing adequate training to healthcare workers (HCWs) are fundamental for effective IPC programs. Compliance with IPC measures significantly affects patient and staff safety as well as the patient care environment [4]. It has been demonstrated that adherence to good IPC practices and procedures by HCWs who received proper education and training is vital for breaking the chain of HAIs by preventing the transmission of resistant organisms to patients and staff members [5–7]. Given the results of the WHO survey, particularly regarding country performance in terms of IPC education and training, this study takes a closer look at the situation in the WHO’s EMR. The study aims to map the IPC education and training components in the different member states of the WHO’s EMR at the national, academic, and healthcare institutional levels. The study addresses the presence of IPC education and training curricula, IPC training programs, profiles of IPC professionals, including physicians and nurses, and type of IPC training opportunities available. The study aims to identify gaps and opportunities in this area and provide suggestions for improving and sustaining regional IPC education and training.

## Methods

### Study design, participants, and recruitment

We conducted purposeful sampling to identify and recruit relevant participants from the pool of national IPC focal persons of each country in the WHO’s EMR. A national IPC focal person is the lead IPC practitioner working at the Ministry/Department of Health within the respective country [8], who is a liaison between the Ministry/Department of Health and the WHO’s country office [8]. Their responsibilities include overseeing the development, implementation, coordination, and evaluation of the national IPC program and its activities [8]. They are also tasked with supporting educational interventions and learning environments to address gaps in IPC workers’ knowledge, skills, and competencies [8].

Participants were invited by email to complete a self-administered online questionnaire between February and March 2023. Consent was obtained through responses to the email. After obtaining consent, participants could complete the survey independently or participate in a virtual meeting with the principal investigator (Rima Moghnieh). This study ensured the collection of nonidentifiable personal information from participants, and the necessary approvals were obtained from the ethics committee of Makassed General Hospital, Beirut, Lebanon (approval number: 222,023).

### Study instrument, data collection, and analyses

To gather data, we employed a self-administered structured questionnaire comprising 13 close-ended questions (with subquestions where necessary) and 1 open-ended question (Supplementary Material 1). The questionnaire was designed based on the WHO’s “Core Component 3” of IPC programs at the national and acute healthcare facility (HCF) levels (IPC education and training) [3, 9, 10]. The tool was written in English and validated by two senior IPC professionals from two countries within the WHO’s EMR.

The closed-ended questions addressed the presence of IPC education and training programs and curricula at the national and institutional levels, profiles of IPC professionals, including physicians and nurses, and format of IPC training opportunities. Multiple questions in the survey covered each of these points. Supplementary Material 2 contains a grid linking each point to specific questions in the survey. The open-ended question sought the personal opinions of focal persons from each country about necessary improvements to IPC education and training in their respective country.

After gathering responses from the principal investigator, the information was compiled and presented quantitatively. Responses were graded with 1 or 0 points for each question based on context, with the total number of participants serving as the denominator. Data analysis was conducted using Microsoft Excel version 16.64 (Microsoft Corporation, Washington, USA).

Following primary data analysis, general trends were identified, and participating countries were categorized into different models based on their IPC education and training approach. The categorization was based on the extent to which the “Multimodal Implementation Strategies,” listed as a core component of effective IPC programs according to WHO Guidelines [3], were applied to IPC education. The focus was on the availability of national IPC guidelines, education, auditing, and corrective actions. A representative country was selected for each country model, and its IPC focal person was invited to comprehensively describe their country’s IPC education and training situation. This section covered key aspects such as the involvement of the national IPC team, if available, in planning, delivering, and supervising IPC training at all levels; the relation between national IPC guidelines and training; the availability of IPC modules in undergraduate education for human health-related specialties offered by academic institutions; the availability of postgraduate education for physicians, nurses, and other healthcare professionals offered by academic institutions; and identified needs for improvement.

## Results

### Education and training components for ipc among participating countries

A total of 15 IPC focal persons from 14 of the 22 EMR countries (Fig. 1) participated in this study. The 14 countries were Afghanistan, Bahrain, Iraq, Jordan, Kuwait, Lebanon, Oman, Pakistan, the occupied Palestinian territories (Gaza Strip and The Westbank), Qatar, Sudan, Syria, United Arab Emirates, and Yemen. The primary mapping of IPC education and training components in these countries is presented in Table 1.

**Table 1:** Mapping the components of the IPC education and training in countries of WHO Eastern Mediterranean Region who accepted to participate in the current study

Component	Total (n = 14^a^)
IPC education and training curricula	
Undergraduate curricula of health sciences majors include defined and elaborate IPC education ^b^	3 (21.4%)
Postgraduate degrees in IPC are available	6 (42.9%)
Training programs are homogenous across the country	8 (57.1%)
Training programs when available are based on National IPC Guidelines put with the support of WHO rather than with academic institutions based on academic curricula	9 (64.3%)
IPC training programs	
National training programs are available for IPC physicians and other professionals	10 (71.4%)
National general healthcare worker and link nurses training programs	5 (35.7%)
Healthcare facility-level healthcare worker (physicians, nurses, others) and link nurses training programs are delivered	13 (92.9%)
Physician IPC professional profile and training	
IPC physicians should be specialized in Infectious Diseases or Medical Microbiology or Community Medicine	8 (57.1%)
Physicians (from any specialty) can become IPC professionals if they receive training upon enrollment prior to practicing	6 (42.9%)
Shortage of Infectious Diseases specialists or Medical Microbiologists	8 (57.1%)
Non-Physician (Nursing) IPC professional profile and training	
Only IPC professionals (nurses) with IPC subspecialty are employed	8 (57.1%)
IPC professionals receive education and training prior to recruitment and/or periodically	10 (71.4%)
Format of IPC training opportunities	
Based on national IPC guidelines	9 (64.3%)
Non-Governmental Organization training courses	11 (78.6%)
Training provided by Scientific Societies	4 (28.6%)
Online training modules	10 (71.4%)

**Abbreviations**:, **IPC**: Infection prevention and control, **WHO**: World Health Organization

**N.B.**

^a^ Included Afghanistan, Bahrain, Iraq, Jordan, Kuwait, Lebanon, Oman, Pakistan, Palestinian Territories, Qatar, Sudan, Syrian Arab Republic, United Arab Emirates, and Yemen

^b^ Limited and unstructured IPC education is available in some undergraduate health majors curricula, in the form of subsections of certain modules, in 7 countries (50%)

Concerning undergraduate IPC education, most countries have dispersed and nonhomogeneous IPC education programs within human health majors. However, this education is not treated as a standalone module; it is rather integrated into the policies and procedures of other modules (Table 1). Academic institutions are seldom involved, whereas elaborate and predefined undergraduate IPC education provided by universities is present in 21.4% of the countries. Less than half of the countries offer postgraduate education opportunities like master’s or Ph.D. degrees in IPC (42.9%) (Table 1).

However, postgraduate professional training targeting IPC physicians and nurses is provided by national IPC teams in 71.4% of these countries. It involves structured training primarily based on national IPC guidelines developed with the WHO’s assistance in 64.3% of the countries (Table 1).

In 92.9% of the countries, the training of HCWs is less uniform and primarily based on healthcare facilities (HCFs) rather than a national training program (Table 1).

Supervision of IPC training is not consistently carried out across the countries. When present, it’s often confined to the context of HCF accreditation or indirectly inferred from IPC key performance indicators, such as hand hygiene compliance rates.

A detailed view of the educational qualifications and prerequisite training profiles of IPC professionals from the participating countries is presented in Table 2.

**Table 2:** Mapping IPC professionals’ profiles and training opportunities in countries of WHO Eastern Mediterranean Region who accepted to participate in this study

Country	IPC physician education profile and training				IPC nurse education profile and training			
	Specialization in ID or Medical Microbiology required	National training programs	Healthcare facility-based training	NGO-facilitated training programs	IPC certification required	National training programs	Healthcare facility-based training	NGO-facilitated training programs
Afghanistan	No (IPC training after appointment)	Yes	Yes	Yes	No (IPC training after appointment)	Yes	Yes	Yes
Bahrain	No (IPC training after appointment)	No	Yes	No	Yes	No	Yes	No
Iraq	No (IPC training after appointment)	Yes	Yes	Yes	No (IPC training after appointment)	Yes	Yes	Yes
Kuwait	Yes	Yes	Yes	No	No (IPC training after appointment)	Yes	Yes	No
Jordan	No (IPC training after appointment)	Yes	Yes	Yes	Yes	Yes	Yes	Yes
Lebanon	Yes	No	Yes	Yes	No (IPC training after appointment)	No	Yes	Yes (+ scientific societies’ training courses)
Oman	Yes	Yes	No	Yes	Yes	Yes	No	Yes (+ scientific societies’ training courses)
Pakistan	Yes	Yes	Yes	Yes	Yes	Yes	Yes	Yes (+ scientific societies’ training courses)
Palestinian Territories	Yes	Yes	Yes	Yes	Yes	Yes	Yes	Yes
Qatar	Yes	Yes	Yes	No	No (IPC training after appointment)	Yes	Yes	No
Sudan	No (IPC training after appointment)	Yes	Yes	Yes	Yes	Yes	Yes	Yes (+ scientific societies’ training courses)
Syrian Arab Republic	No (IPC training after appointment)	Yes	Yes	Yes	No (IPC training after appointment)	Yes	Yes	Yes
United Arab Emirates	Yes	No	Yes	No	Yes	No	Yes	Yes
Yemen	Yes	Yes	Yes	Yes	Yes	Yes	Yes	Yes

**Abbreviations**: **ID**: Infectious Diseases, **IPC**: Infection prevention and control, **WHO**: World Health Organization

In 57.1% of these countries, a shortage of infectious disease (ID) physicians or medical microbiologists is observed despite the requirement that IPC physicians must specialize in ID or medical microbiology (Tables 1 and 2). In 42.9% of the countries, practicing IPC physicians are medical doctors from outside the mentioned specialties and require IPC training prior to employment and nonphysician IPC professionals, such as IPC nurses, are not required to specialize in IPC; however, they must attend training sessions before starting practice and periodically thereafter. IPC nurses receive education and training before recruitment and/or periodically in 71.4% of the countries.

In this region, nongovernmental organizations (NGOs) such as the WHO play a significant role in IPC education and training, mainly through train-the-trainer programs and assisting the country’s IPC authorities in establishing national IPC guidelines (78.6%). The NGOs themselves often fund this training. However, scientific societies provide IPC training in only 28.6% of the countries.

### Proposed country model for infection prevention and control education and training among participating countries

Based on these survey results (Tables 1 and 2), we identified three country models of IPC education and training from the participating countries:

- Model 1: HCF-Based Education and Training.
- Model 2: National IPC Guidelines-Based Education and Training.
- Model 3: National and HCF-Based IPC Education and Training in Collaboration with Academic Institutions.

#### Model 1: HCF-based education and training

IPC education and training in this model (namely Lebanon) predominantly rely on HCFs rather than on a national education program.

- At the national level, an endorsed national action plan on AMR by the Lebanese Ministry of Public Health (MoH) has existed since 2019, with one of its objectives dedicated to IPC. An AMR steering committee oversees its implementation, although this process was halted due to the COVID-19 pandemic.
- An IPC program exists under the national AMR plan’s umbrella, led by the Preventive Medicine Department (PMD) at the MoH. However, there is no independent national IPC team. PMD handles IPC training activities in collaboration with international NGOs. During the COVID-19 pandemic, ad hoc training sessions for HCWs were conducted by the MoH and WHO, along with the private sector, focusing on personal protective equipment and transmission-based precautions.
- IPC practices are central to mandatory standards in HCF accreditation audits. Accreditation or reaccreditation occurs every four to five years, assessing IPC practices at the national level. IPC education is part of general HCW training as per IPC practices’ national standards within HCFs. It is recommended that an ID physician lead the HCF’s IPC team, supported by an IPC officer with a Bachelor of Science in nursing or medical laboratory.
- National IPC guidelines are available; however, IPC education and practice rely on international guidelines from sources such as the WHO, European Committee on Infection Control (EUCIC), Association for Professionals in Infection Control and Epidemiology (APIC), U.S. Centers for Disease Control and Prevention (CDC), Society for Healthcare Epidemiology of America, and others.
- Despite the lack of a standardized curriculum, HCFs play an important role in IPC implementation and training. IPC physicians and professionals prepare their training material at the institutional level. General HCWs’ IPC training occurs within the HCF and is led by its IPC team. Some HCFs offer IPC education for all staff upon employment and then periodically, although this varies across HCFs. Nonetheless, all HCFs provide basic IPC education, covering standard precautions, transmission-based precautions, and IPC bundles for nosocomial infections.
- Undergraduate education briefly addresses general community-based hygiene, without a dedicated IPC module.
- IPC is not a distinct specialty for physicians. It is incorporated into ID practical training but lacks formal organization or structure. ID physicians invest personal efforts in IPC education. IPC certification is not mandatory for hiring ID physicians as IPC team leaders in HCFs.
- Nonphysician IPC professionals, such as IPC nurses, receive on-the-job training guided by IPC ID physicians. They also attend train-the-trainer courses by organizations such as the WHO, Lebanese Society of Infectious Diseases and Clinical Microbiology, National Syndicate of Nurses, or HCFs themselves. Few IPC professionals in Lebanon hold the Certification in Infection Prevention and Control (CIC) from the U.S. Certification Board of Infection Control and Epidemiology, a personal choice rather than a recommended prerequisite.
- Learning opportunities exist in Lebanon, but they are not standardized or coordinated. Some universities offer a master’s degree in IPC for nurses. Lebanese IPC professionals often attend international seminars and conferences in person or online. Several ID physicians and medical microbiologists attend European Congress of Clinical Microbiology and Infectious Diseases and IDWeek, offering diverse IPC sessions to enhance professionals’ knowledge and skills.
- To improve IPC education and training, strengthening national IPC education is crucial, monitored by a national IPC team. This would ensure consistent, quality, and quantifiable education across the country. An organized, well-coordinated system guarantees sustainable IPC education across private and public healthcare sectors, alongside individual efforts. A national curriculum for different professionals, ranging from undergraduate majors to specialized courses and degrees for IPC professionals, is essential. Establishing IPC as a standalone specialty for physicians and microbiologists is also needed, driven by job opportunities in IPC-related research for financial and scientific sustainability.

#### Model 2: National IPC guideline-based education and training

The national IPC team organizes and delivers most IPC education and training in this model.

- The national IPC team conducts training for IPC professionals and for other HCWs in some cases, often in the form of workshops. This training is typically mandatory upon employment of IPC professionals and aligns with national IPC guidelines.
- Education and training outside nationally organized training are not supervised by the national IPC team. IPC key performance indicators, such as hand hygiene compliance, reflect this training. With the COVID-19 pandemic, awareness and interest in IPC among healthcare providers, including senior administrators, have increased.
- In several of the WHO’s EMR countries adopting this model, national IPC guidelines were developed with the support of the WHO, forming the basis for IPC training. The national IPC guidelines draw from international guidelines adapted to local contexts. National IPC teams are responsible for educating and training IPC professionals. Academic institutions and universities have limited involvement in this process.
- Undergraduate IPC education in health sciences varies and is not a standalone module. Instead, it is integrated into other majors such as dentistry or medical laboratory. Moreover, it is not a general course across all human health-related studies.
- In several EMR countries adopting this model, becoming an IPC professional requires enrolling in educational and train-the-trainer courses offered by the national IPC team for physicians and nurses. General HCWs receive training at the facility level from local IPC professionals upon employment and periodically after that, based largely on national IPC guidelines. IPC training by HCFs lacks a defined curriculum and is seldom standardized or synchronized among HCFs. Nevertheless, HCFs accredited by international bodies, such as the Joint Commission International, often offer structured IPC education, reflected in IPC key performance indicators; however, the national IPC team does not monitor them.
- Limited opportunities for higher education in IPC exist for IPC professionals and rarely for IPC physicians.
- Enhancing IPC education planning and implementation in these countries is crucial, thereby reducing dependence on NGO assistance. Job opportunities should incentivize IPC specialization among professionals, fostering higher-level IPC education. This would facilitate local mentors in IPC and necessitate standardized national IPC education curricula. Universities should actively participate in IPC education and curricula development, thereby boosting awareness regarding IPC’s significance among policymakers, administrators, politicians, and intellectuals.

#### Model 3: National and HCF-based ipc education and training in collaboration with academic institutions

- This country model (Sultanate of Oman) entails a joint effort by the national IPC team, HCFs, and academic institutions.
- IPC education and training in Oman primarily focus on nursing through the national diploma program. Physicians, typically ID specialists or clinical microbiologists, receive IPC training through their curriculum. The national infection prevention continuous education program recently incorporated internationally experienced training parties, such as the Infection Control Africa Network (ICAN) and APIC for IPC professionals’ training and certification.
- IPC training for health students and HCWs was initially part of HCF programs. However, a 2020 ministerial declaration mandated this training for all health students and workers before clinical training or practice, renewed every three years.
- A manual for this training was prepared by national programs, covering theory and competency assessment (hand hygiene and personal protective equipment). Certification for passing basic IPC training is awarded upon completion.
- Health students’ full program is overseen by their training institution, following core elements in the national program guideline, ensuring training and certification before clinical training. IPC training and certification for all HCWs involve an online course covering theoretical aspects and case studies, followed by competency training and assessment by the IPC team at the HCFs. This course was designed and provided in collaboration with the Omani Medical Specialty Board.
- Oman’s IPC training system adapts to service and program needs. Many infection preventionists pursue senior leadership positions in quality, health administration, health education, training, and public health units.

### Future needs and suggestions

Most participants believe IPC education and training need to shift from ad hoc to an academic standalone specialty (Table 3). Standardizing IPC training across different HCFs in the same country is also a common demand, alongside a regional IPC training curriculum tailored to the region’s needs. Several IPC focal persons consistently highlighted the shortage of ID physicians and medical microbiologists in their countries. Creating job opportunities for such specialists could attract new-generation physicians and microbiologists to these fields. Increasing the number and enhancing the qualifications of IPC professionals would encourage the growth of local or regional scientific societies involved in IPC training, education, and collective IPC-oriented research.

**Table 3:** Current IPC education and training improvement needs in countries of WHO Eastern Mediterranean Region who accepted to participate in this study

Country	Training Needs
Afghanistan	• Strengthen and create focused IPC education modules in undergraduate health sciences majors. University specialties and higher degrees in IPC with the help of WHO. • Design and provide different training programs to different categories of HCWs. • Competency training outside the country and exposure to other experienced countries • Need for well-equipped centers (for simulation)
Bahrain	• Strengthen and create focused IPC education modules in undergraduate health sciences majors. University specialties and higher degrees in IPC. • Involve academic institutions in designing IPC curriculum and national IPC guidelines. • Make IPC education autonomous in the countries, rather than dependent NGO support by integrating IPC education into national health education plans. • National supervision of IPC education and training. • Design and provide different training programs to different categories of HCWs. • Provide a regional advanced IPC degree, supported by leading academic institutions in the region. • Make IPC training mandatory for all HCW with periodic licensing and relicensing.
Iraq	• Training of hospital administrators
Kuwait	• Strengthen and create focused IPC education modules in undergraduate health sciences majors. University specialties and higher degrees in IPC. • Design and provide different training programs to different categories of HCWs. • Required qualifications for IPC professionals
Jordan	• University specialties and higher degrees in IPC • Identify qualifications of IPC trainers • Relate IPC to research • Relate the professional development with IPC courses (continuous professional education hours that are related to IPC) • Need for national IPC curriculum
Lebanon	• Put a national definition of IPC certification. • National supervision of IPC education and training. • Need for national IPC curriculum
Oman	• National preparedness for health emergencies to be mandated within the professional career pathways
Pakistan	• Design and provide different training programs to different categories of HCWs. • Need for a program that evaluates training needs
Palestinian Territories	• Strengthen and create focused IPC education modules in undergraduate health sciences majors. University specialties and higher degrees in IPC. • Need for accredited courses in the world regarding infection control, not just local ones • Exposure to regional success stories (successful people with good indicators)
Qatar	• Design and provide different training programs to different categories of HCWs. • International or Regional IPC-certified training supported by WHO • Unified well structures IPC module based on the scope of work (postgraduate)
Sudan	• Strengthen and create focused IPC education modules in undergraduate health sciences majors. University specialties and higher degrees in IPC. • National supervision of IPC education and training.
Syrian Arab Republic	• Develop undergraduate IPC education modules • Develop an IPC residency program • Improve the awareness of administrators and stakeholders on the importance of IPC
United Arab Emirates	• Strengthen and create focused IPC education modules in undergraduate health sciences majors. University specialties and higher degrees in IPC. • Design and provide different training programs to different categories of HCWs.
Yemen	• Adoption of the IPC curriculum prepared by the Ministry of Public Health and Population- Sana’a for the National Training Manual of IPC in undergraduate and postgraduate medical and paramedical majors • Financial support to establish continuous training courses

**Abbreviations**: **HCW**: healthcare worker, **IPC**: Infection prevention and control, **WHO**: World Health Organization

## Discussion

In the current survey involving national focal points for IPC in MoHs or other governmental organizations across 14 countries in the WHO’s EMR, we identified significant variability in terms of IPC education and training levels, formats, and requirements among the participating countries. As previously mentioned, the WHO’s EMR comprises 22 countries and approximately 10% of the world’s population. The intricate socioeconomic situations and geopolitical conflicts have amplified the impact of epidemics in low- to lower-middle-income countries. Most countries have scattered nonhomogeneous IPC education programs in human health undergraduate majors and academic institutions are rarely involved. In 71.4% of these countries, postgraduate training targeting IPC professionals is provided by national IPC teams, primarily based on national IPC guidelines developed with the aid of the WHO. Generally, healthcare worker training relies heavily on healthcare facilities in 92.9% of the countries, rather than on a national training program. A comparison of the IPC education and training description in the third model from Oman with that of other regional models reveals a more structured approach. Oman’s model involves academic institutions and features national IPC curricula that target different HCW groups based on their roles within the healthcare system.

The first WHO global cross-sectional survey on the implementation of IPC core components in HCFs, conducted in 2019, reported findings similar to ours. Tomczyk et al. invited IPC professionals to complete the online WHO IPC Assessment Framework (IPCAF) survey, yielding 4440 responses from 81 countries [11]. Although the overall weighted IPCAF median score indicated an advanced level of core component implementation, significant disparities were observed in the scores of countries of different socioeconomic classes and of each individual component [11]. Notably, lower IPCAF scores were recorded in low-income countries and public facilities compared to high-income countries and the private sector [11]. Core Component 7 (workload, staffing, and bed occupancy) and Core Component 3 (education and training) received the lowest scores [11].

This study found that 64.3% of the 14 countries provide professional IPC training based on national IPC guidelines. The 2021–2022 global survey on IPC minimum requirements at the national level, conducted by the WHO across 22 EMR countries, indicated that 72.7% of these countries mandated their national IPC teams to produce IPC guidelines, and 63.6% of them produced guidelines following evidence and international standards [1]. A previous similar global survey conducted in 2017–2018 revealed that the WHO’s EMR had the lowest frequency of developing national IPC guidelines, at only 50% [12]. Thus, comparing recent data, including ours, with that of the 2017–2018 survey demonstrates an improvement in this critical indicator [1, 12].

Over the past decade, significant investments have been made in low-resource countries to address acute needs, such as the recent Ebola epidemic and the ongoing COVID-19 pandemic. NGOs, such as the WHO, have played a crucial role in these efforts. This role is evident in our current survey, where 78.6% of countries rely on NGOs for national-level IPC education and training and support for HCF-level education through train-the-trainer courses. Moreover, 64.3% of countries implement their IPC guidelines with support from the WHO. Although this form of support is important, the sustainability and longevity of such accomplishments and investments in IPC education and training are vulnerable.

Sustainability largely hinges on integrating IPC principles and practices into higher education programs, rather than relying solely on external support for designing and implementing IPC education curricula. In our survey, academic institutions actively provided comprehensive IPC education within undergraduate health sciences curricula in 21.4% of participating countries only. Conversely, higher education is available in 42.9% of countries. The Lebanese example (Model 1) underscores the role of academic institutions, scientific societies, and NGOs in partially addressing gaps resulting from the absence of a functional national IPC program.

Another crucial factor influencing sustainability is the recognition of IPC as a distinct specialty and the availability of sufficient specialized professionals and mentors for IPC education. In 42.9% of surveyed countries, IPC physicians are not necessarily ID specialists or medical microbiologists. Additionally, 57.1% of countries face a shortage of these medical specialists. Therefore, considering IPC as a standalone specialty could expand education and training opportunities in this field, create more job prospects for specialists, and encourage greater enrollment, ultimately ensuring sustainability. This is particularly pertinent for countries grappling with high HAI and AMR rates. For instance, IPC training for nurses and doctors in Europe varies across countries, encompassing differences in content, format, assessment, and recognition [13]. Other disparities exist at sociocultural, healthcare system, and HAI/AMR epidemiology levels [13]. Addressing this heterogeneity culminated in establishing “The European Infection Prevention and Control Certificate.” This two-year interdisciplinary training program covers clinical microbiology, infection control, hospital hygiene, and IDs and is endorsed by European national professional bodies and the European Society of Clinical Microbiology and Infectious Diseases [13].

In this study, the Omani example, characterized by a robust national IPC program and active involvement of academic institutions in IPC education, serves as an optimal model. It ensures the judicious application of IPC core components, sustains IPC program activity, and facilitates its long-term evolution in response to emerging epidemic situations.

Identifying gaps in IPC education and training by IPC focal persons has led to several suggestions. Firstly, academic institutions need to take a more proactive role in developing IPC education and training curricula, integrating essential principles into undergraduate education as standalone modules within health-related majors, rather than incorporating them solely into various courses. Secondly, academic institutions are encouraged to introduce higher education degrees and diplomas in IPC. Thirdly, creating positions for mentors and academic IPC professionals is recommended. Fourthly, the development of national or regional IPC curricula is crucial. Additionally, a standardized and accredited IPC certification was recognized as a necessity in the EMR.

Countries like Saudi Arabia and Egypt already offer local IPC courses and training programs. In Saudi Arabia, the General Directorate of Infection Prevention and Control has established the mandatory “Basic Infection Control Skills License” program, providing fundamental infection control skills for all HCWs with patient contact. However, this program does not substitute the need for periodic infection control educational programs targeting various HCW categories. In Egypt, private and public sectors offer IPC certification programs, often affiliated with universities like Ain Shams University. The certifications are recognized locally and in neighboring countries such as Sudan and the occupied Palestinian territories.

Similar to the EUCIC certification example, a tailored program could be developed by leading academic institutions across different countries in the EMR. Academic institutions could start by adopting existing international training programs and certifications, such as the EUCIC or the APIC CIC, as templates to create a regional certification program. Such an initiative was successful in Africa, where the ICAN, a prominent African IPC non-profit organization, trained professionals on IPC and antimicrobial stewardship programs at facility and national levels. They established hubs across the continent to ensure uniform access to standardized training. During the COVID-19 pandemic, ICAN extended its activities beyond Africa, offering virtual IPC training to professionals in English- and French-speaking countries. Such a project would ensure equity among IPC professionals regarding employment opportunities in an era of heavy immigration due to political and economic turmoil.

## Limitations

One limitation of this study is that this mapping could not encompass all member states of the EMR, along with the opinions of corresponding national IPC focal points. Another limitation pertains to potential differences in the interpretation of questions, affecting the capture of detailed and nuanced insights from participants. Nonetheless, using a simplified questionnaire offers insights into the situation and requirements for IPC training and education in the region.

## Conclusion

This study underscores IPC education and training disparities across the WHO’s EMR countries. Although national efforts are noticeable in this mapping, some countries still lack national IPC programs and teams, necessitating the pursuit of such initiatives. Establishing a specialized regional scientific network in IPC could effectively bridge the prevailing gaps and standardize IPC education within and across nations. The formulation of regional IPC certification standards and uniform educational curricula is imperative. Additionally, tailored education programs targeting distinct categories of HCWs based on their level of involvement in IPC are essential. Lastly, the need for national assessment and governance of IPC education at the country level remains crucial.

### Electronic supplementary material

Below is the link to the electronic supplementary material.

Supplementary Material 1

Supplementary Material 2

## Data Availability

The data that support the findings of this study will be shared on reasonable request to the corresponding author.

## Abbreviations

AMR: Antimicrobial Resistance
APIC: Association for Professionals in Infection Control and Epidemiology
CIC: Certification in Infection Prevention and Control
COVID-19: Coronavirus Disease 2019
ECCMID: European Congress of Clinical Microbiology and Infectious Diseases
EMR: Eastern Mediterranean Region
ESCMID: European Society of Clinical Microbiology and Infectious Diseases
EUCIC: European Committee on Infection Control
HAI: Healthcare-Associated Infections
HCF: Healthcare Facilities
HCWs: Healthcare Workers
ICAN: Infection Control Africa Network
ID: Infectious Diseases
IPC: Infection Prevention and Control
IPCAF: Infection Prevention and Control Assessment Framework
MoH: Ministry of Health
NGO: Non-governmental Organization
WHO: World Health Organization
