# Supplementary material for: Mapping of infection prevention and control education and training in some countries of the World Health Organization’s Eastern Mediterranean Region: current situation and future needs
Source: Antimicrob Resist Infect Control. 2023 Sep 4;12:90. doi: 10.1186/s13756-023-01299-9 (PMC10478460; doi:10.1186/s13756-023-01299-9)
Supplement: Supplementary file 1 — Supplementary Material 1 [file 13756_2023_1299_MOESM1_ESM.pdf]

Infection prevention and control (IPC) education and training in countries of the WHO EMRO: Mapping the current situation and future needs

Questionnaire:

1. IPC Undergraduate education
   1.1. Is IPC (courses, modules, etc) included in undergraduate education of medical/dental and paramedical specialties (nursing, pharmacy, laboratory, etc)? Yes/No

   1.2. Is this education related to IPC among educational institutions homogenous/similar, i.e. one similar curriculum exists? Yes/No

   1.3. Are there national IPC undergraduate curricula for the different medical and paramedical specialties that are developed in collaboration with local academic institutions, and adopted by them? Yes/No

   1.4. Are these national IPC undergraduate curricula currently taught to undergraduate students in the educational facilities? Yes/No

2. IPC Postgraduate education
   2.1. Is IPC being taught as a stand-alone subspecialty? Yes/No
   2.2. Are there higher education degrees MSc or PhD in IPC available? Yes/No
   2.3. Are there national IPC postgraduate and in-service curricula that are developed in collaboration with local academic institutions for IPC professionals:
      2.3.1. IPC physician Yes/No
      2.3.2. IPC non-physician (nurses, etc.) Yes/No
   2.4. Is there a national recognition for IPC postgraduate programs? (Are they mentioned in accreditation criteria, job description of IPC professionals, etc.)? Yes/No

3. Postgraduate training opportunities and availability for different medical and paramedical healthcare professionals
   3.1. For physicians
      3.1.1. At the national level, Yes/No
      3.1.2. At the healthcare facility level, Yes/No
   3.2. For link nurses or champions
      3.2.1. At the national level, Yes/No
      3.2.2. At the healthcare facility level, Yes/No
   3.3. For nurses, pharmacists, laboratory personnel and other healthcare workers
      3.3.1. At the national level, Yes/No
      3.3.2. At the healthcare facility level, Yes/No

4. Is it mandatory to have an IPC team in every healthcare facility? Yes/No

5. Is healthcare facility training homogenous among all healthcare facilities in the country? Yes/No

6. Is this healthcare facility training based on a standardized national curriculum? Yes/No

7. Education requirements of IPC professionals in healthcare facilities:
   7.1. IPC physicians should be specialized in ID or medical microbiology or other physicians can be IPC physician, no need for further certification. Yes/No
   7.2. IPC physicians should have a certified training in IPC . Yes/No
   7.3. IPC nurses are requested to be specialized in IPC (have a degree in IPC or followed a nationally recognized official training). Yes/No
   7.4. Any competent nurse can be employed as IPC professional. Yes/No

8. Training availability and format for IPC professionals (nurses and physicians):
   8.1. At the national level, Yes/No
   8.2. At the healthcare facility level, Yes/No
   8.3. Provided by NGOs like WHO, UNICEF, or others, Yes/No
   8.4. Online training, Yes/No
   8.5. Funded training, Yes/No
   8.6. Self-learning on the job, Yes/No

9. Training format for IPC nurses
   9.1. National periodic training courses Yes/No
   9.2. Structured scientific societies training courses Yes/No
   9.3. NGO training courses. Yes/No

10. Training format for other healthcare workers
    10.1. At the national level, Yes/No
    10.2. At the healthcare facility level, Yes/No
    10.3. Single seminar/lecture, Yes/No
    10.4. Upon employment only, Yes/No
    10.5. Upon employment and periodic, Yes/No

11. Are IPC courses/seminars available by scientific societies in the country? Yes/No

12. Are IPC courses/seminars available by NGOs in the country? Yes/No

13. What do you think are the local needs for IPC education and training at all levels?
