# Supplementary material for: Mapping of infection prevention and control education and training in some countries of the World Health Organization’s Eastern Mediterranean Region: current situation and future needs
Source: Antimicrob Resist Infect Control. 2023 Sep 4;12:90. doi: 10.1186/s13756-023-01299-9 (PMC10478460; doi:10.1186/s13756-023-01299-9)
Supplement: Supplementary file 2 — Supplementary Material 2 [file 13756_2023_1299_MOESM2_ESM.docx]

**Supplementary Material**

**Components of the IPC education and training in countries of WHO Eastern Mediterranean Region derived from the study questionnaire**

|  | **Questions** |
| --- | --- |
| **IPC education and training curricula** |  |
| Undergraduate curricula of health sciences majors include defined and elaborate IPC education | 1 (1.1 to 1.4) |
| Post-graduate degrees in IPC are available | 2.1 and 2.2 |
| Training programs are homogenous across the country | 2.3 and 5 |
| Training programs when available are based on National IPC Guidelines put with the support of WHO rather than with academic institutions based on academic curricula | 1.4, 2.3, and 2.4 |
| **IPC training programs** |  |
| National training programs are available for IPC physicians and other professionals | 3.1, 3.3, and 8.1 |
| National general healthcare worker and link nurses training programs | 3.2 |
| Healthcare facility-level healthcare worker (physicians, nurses, others) and link nurses training programs are delivered | 3 and 8 |
| **Physician IPC professional profile and training** |  |
| IPC physicians should be specialized in Infectious Diseases or Medical Microbiology or Community Medicine | 7.1 |
| Physicians (from any specialty) can become IPC professionals if they receive training upon enrollment prior to practicing | 7.2 |
| Shortage of Infectious Diseases specialists or Medical Microbiologists | 7.1 |
| **Non-Physician (Nursing) IPC professional profile and training** |  |
| Only IPC professionals (nurses) with IPC subspecialty are employed | 7.3 and 7.4 |
| IPC professionals receive education and training prior to recruitment and/or periodically | 9 and 10 |
| **Format of IPC training opportunities** |  |
| Based on national IPC guidelines | 8, 9, and 10 |
| Non-Governmental Organization training courses | 8,9, and 12 |
| Training provided by Scientific Societies | 9 and 11 |
| Online training modules | 8 |
